# True mol­ecular conformation and structure determination by three-dimensional electron diffraction of PAH by-products potentially useful for electronic applications

**DOI:** 10.1107/S205225252201154X

**Published:** 2023-01-01

**Authors:** Iryna Andrusenko, Charlie L. Hall, Enrico Mugnaioli, Jason Potticary, Simon R. Hall, Werner Schmidt, Siyu Gao, Kaiji Zhao, Noa Marom, Mauro Gemmi

**Affiliations:** a Center for Material Interfaces, Electron Crystallography, Instituto Italiano di Tecnologia, Pontedera 56025, Italy; b School of Chemistry, University of Bristol, Bristol BS8 1TS, United Kingdom; c Department of Earth Sciences, University of Pisa, Pisa 56126, Italy; d PAH Research, Igling-Holzhausen, D-86859, Germany; e Department of Materials Science and Engineering, Carnegie Mellon University, Pittsburgh, Pennsylvania 15213, USA; University of California, Los Angeles, USA

**Keywords:** 3D electron diffraction, structure determination, polycyclic aromatic hydro­carbons, density functional theory, many-body perturbation theory, singlet fission, triplet–triplet annihilation, crystal structure

## Abstract

The true mol­ecular conformation and the crystal structure of benzo[*e*]di­naphtho­[2,3-*a*;1′,2′,3′,4′-*ghi*]fluoranthene, 7,14-di­phenyl­naphtho­[1,2,3,4-*cde*]bis­anthene and 7,16-di­phenyl­naphtho­[1,2,3,4-*cde*]heli­anthrene were determined *ab initio* by 3D electron diffraction. All three mol­ecules are remarkable polycyclic aromatic hydro­carbons. The mol­ecular conformation of two of these com­pounds could not be determined *via* classical spectroscopic methods due to the large size of the mol­ecule and the occurrence of multiple and reciprocally connected aromatic rings. The mol­ecular structure of the third mol­ecule was previously considered provisional. These com­pounds were isolated as by-products in the synthesis of similar products and were at the same time nanocrystalline and available only in very limited amounts. 3D electron diffraction data, taken from submicrometric single crystals, allowed for direct *ab initio* structure solution and the unbiased determination of the inter­nal mol­ecular conformation. Detailed synthetic routes and spectroscopic analyses are also discussed. Based on many-body perturbation theory simulations, benzo[*e*]di­naphtho­[2,3-*a*;1′,2′,3′,4′-*ghi*]fluoranthene may be a promising candidate for triplet–triplet annihilation and 7,14-di­phenyl­naphtho­[1,2,3,4-*cde*]bis­anthene may be a promising candidate for inter­molecular singlet fission in the solid state.

## Abstract 2

The true mol­ecular conformation and the crystal structure of three remarkable polycyclic aromatic hydro­carbons were determined *ab initio* by 3D electron diffraction, which allowed for direct *ab initio* structure solution and the unbiased determination of the inter­nal mol­ecular conformation. Detailed synthetic routes and spectroscopic analyses are also discussed.

## 1. Introduction

Polycyclic aromatic hydro­carbons (PAHs) are organic mol­ecules containing a series of conjugated carbon rings. The term ‘PAH’ is generally restricted to com­pounds consisting of only C and H atoms, and com­prising two or more aromatic rings bonded in various arrangements (Lawal, 2017 ▸). Early syntheses of PAHs (Anschütz, 1886 ▸; Scholl *et al.*, 1910 ▸; Scholl & Meyer, 1932 ▸) attracted considerable inter­est due to their opto­electronic properties, which led to the creation of a number of functionalized com­pounds based on PAH backbones (Buchlovič *et al.*, 2013 ▸; Ko *et al.*, 2018 ▸; Castro *et al.*, 2019 ▸). In particular, in the mid-1900s, Clar and co-workers synthesized and identified a number of PAHs and related com­pounds with unique physical and chemical properties (Clar *et al.*, 1964 ▸, 1981 ▸; Clar & Schmidt, 1975 ▸).

In most cases, the identity of the newly synthesized organic com­pounds can be determined from combustion analysis and by a combination of NMR, IR, UV and mass spectrometry. In particular, a com­parison of the UV and photoelectron (PE) spectral data provides information about the purity and helps to distinguish ‘isotopic PAHs’, *i.e.* com­pounds with different shapes but identical UV band positions (Clar & Schmidt, 1976 ▸, 1979 ▸; Clar *et al.*, 1981 ▸).

It is often impossible to characterize PAHs by single-crystal X-ray diffraction (XRD) because of the difficulty in obtaining suitable sufficiently large crystals. Therefore, the final confirmation is generally attained by powder XRD analysis. How­ever, when there are many possible mol­ecular structures that can match a particular stoichiometry, it may be difficult to determine the exact conformation based on spectroscopic data alone. Furthermore, PXRD is intrinsically limited to one-dimensional data and hampered by the overlap of independent reflections, which may become rather severe for unit-cell parameters longer than 20 Å. As a consequence of this unsatisfactory state of affairs, the conformation of several large PAHs mentioned in the literature is uncertain or unknown, and many more are considered suspect (Clar *et al.*, 1981 ▸; Fetzer, 2000 ▸, 2007 ▸; Wilkes, 2010 ▸).

In recent years, electron diffraction (ED) has become a routine option for crystal structure determination when crystals sufficiently large for single-crystal XRD cannot be grown. The key development was the establishment of procedures for collecting and recombining 3D electron diffraction (3D ED) data (Kolb *et al.*, 2007 ▸; Wan *et al.*, 2013 ▸; Gemmi *et al.*, 2019 ▸; Nannenga & Gonen, 2019 ▸). The 3D ED method has been used for the structure determination of hundreds of previously unknown structures, including inorganic materials (Kaiukov *et al.*, 2020 ▸; Krysiak *et al.*, 2021 ▸; Kapaca *et al.*, 2021 ▸), small-mol­ecule organics (Andrusenko *et al.*, 2019 ▸; Jones *et al.*, 2019 ▸; Brázda *et al.*, 2019 ▸; Cui *et al.*, 2020 ▸; Levine *et al.*, 2020 ▸; Bruhn *et al.*, 2021 ▸; Hall *et al.*, 2021 ▸; Andrusenko *et al.*, 2021 ▸; Papi *et al.*, 2021 ▸), peptides and macromolecules (Sawaya *et al.*, 2016 ▸; Krotee *et al.*, 2018 ▸; Lanza *et al.*, 2019 ▸; Xu *et al.*, 2019 ▸; Warmack *et al.*, 2019 ▸). It is noteworthy that *ab initio* crystal structure determination does not require information about the mol­ecular conformation, but only a rough estimation of the atomic content of the unit cell. In this article, we report the true mol­ecular conformation and the crystal structure determined by the 3D ED method for three PAHs belonging to two different systems.

**Figure 1:**
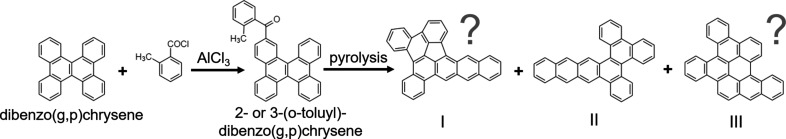
A stepwise model of the synthesis of benzo[*e*]di­naphtho­[2,3-*a*;1′,2′,3′,4′-*ghi*]fluoranthene (I) and related products (II and III). The mol­ecule of I was considered provisional before its structure determination by 3D ED, whereas the mol­ecular structure of III is still speculative.

Fluoranthenes are one of the most studied classes of PAHs (Haritash & Kaushik, 2009 ▸). They are also known as ‘non-alternant PAHs’ because they contain six-membered benzene rings fused with an additional five-membered ring (Gupte *et al.*, 2016 ▸). Fluoranthene-based PAHs serve as a basic unit in preparing chromophores for organic light-emitting diodes (OLEDS) and field-effect transistors (Saranya *et al.*, 2011 ▸). Benzo[*e*]di­naphtho­[2,3-*a*;1′,2′,3′,4′-*ghi*]fluoranthene (I) is form­ed by pyrolysis together with two other hydro­carbons, namely, benzo[*a*]phenanthro[9′,10′-*c*]tetra­cene (II) and di­benzo[*a*,*k*]naphtho[1,2,3,4-*ghi*]perylene (III). Fig. 1 ▸ illustrates a stepwise model of the corresponding synthetic route des­cribed by Clar and co-workers (Clar *et al.*, 1964 ▸). The mol­ecular conformation of II is supported by several spec­tro­scopic data, its reactive behaviour and, most conclusively, by an alternative synthesis with Zn dust that produces II exclusively (Fig. S1). Conversely, the mol­ecular conformations of I and III were only reported as tentative (Clar *et al.*, 1964 ▸). Spectroscopic data for II and III are presented and discussed in the supporting information.

**Figure 2:**
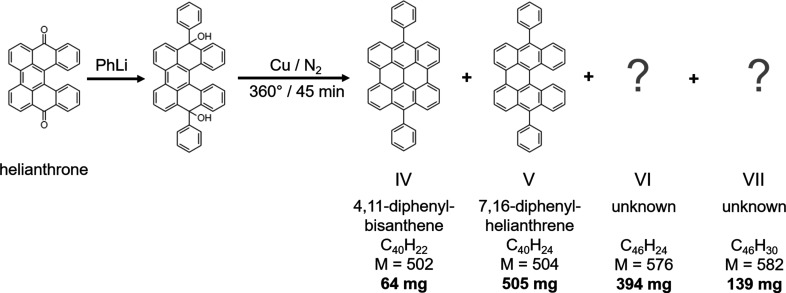
A stepwise model of the synthesis of 4,11-di­phenyl­bis­anthene (IV). The by-product V was identified and determined in Arabei & Pavich (2004 ▸). The mol­ecular conformation and crystal structure of by-products VI and VII were unknown prior to their determination by the 3D ED method. For each product of the reaction, the formula, mol­ecular weight (*M*) and yield (in bold) are reported.

The second system studied in this article involves both bis­anthene and heli­anthrene com­pounds. Initially, 4,11-di­phenyl­bis­anthene (IV) was synthesized by Sauvage, who only determined the maximum positions of the longwave absorption bands (Sauvage, 1947*a* ▸,*b* ▸). Renewed inter­est in this com­pound was sparked in the 1970s, as it was shown to be one of the few mol­ecules to fluoresce at IR wavelengths (Maulding, 1970 ▸; Rauhut *et al.*, 1975 ▸). Following the procedure of Sauvage’s synthesis (Sauvage, 1947*a* ▸,*b* ▸), we attempted to synthesize new IR emitters with a high fluorescent quantum yield starting from helianthrone. Fig. 2 ▸ illustrates a stepwise model of the corresponding synthetic route. During this reaction, in addition to 4,11-diphenylbis­anthene (IV), a number of by-products occur, among which only 7,16-di­phenylheli­anthrene (V) was identified previously (Arabei & Pavich, 2004 ▸). This com­pound appears in a greater yield when N_2_ is bubbled through the system during the reaction (Fort, 2010 ▸). Two additional by-products of the reaction, VI and VII, were not identified *via* spectroscopic data, but appear to form *via* a Diels–Alder condensation reaction. The mol­ecular structures of by-products VI and VII have hitherto remained unknown, due to the large size of the mol­ecules and the occurrence of multiple and reciprocally connected aromatic rings.

Here, we present the *ab initio* structure determination of com­pounds I, VI and VII, obtained through 3D ED data. This method also allowed for the confirmation of the predicted mol­ecular conformation for com­pound I and for the true mol­ecular identification of com­pounds VI and VII. Moreover, detailed synthetic routes and spectroscopic analyses of all the synthetic products are reported. Having solved the structures of these com­pounds, we eventually use many-body perturbation theory calculations within the GW approximation and the Bethe–Salpeter equation (BSE) (Golze *et al.*, 2019 ▸; Blase *et al.*, 2020 ▸) to assess their potential usefulness in photovoltaics.

## 2. Methods

### 2.1. Synthesis

Samples I, II and III were obtained starting from a Friedel–Crafts reaction (Friedel & Crafts, 1877 ▸) between dibenzo[*g*,*p*]chrysene (10 g) and *o*-toluyl chloride, with AlCl_3_ as catalyst. The two reactants were connected by a ketone group, either 2- or 3-(*o*-tolu­yl)dibenzo[*g*,*p*]chrysene (12 g). The 1- and 4-positions are excluded on steric grounds. After Elbs pyrolysis (15 g, 420–430 °C, 10 min under CO_2_), fractional crystallization and chromatography showed that three products had formed (Fig. 1 ▸): I (0.46 g, violet–red needles, m.p. 348–350 °C), II (1.5 g, thick orange–yellow needles, m.p. 258–259 °C) and III (0.1 g, fibrous orange–yellow needles, m.p. 282°C).

Multiple syntheses of samples IV, V, VI and VII (Fig. 2 ▸ and Fig. S2) started from adding phenyl–Li to helianthrone, which can be prepared on a 100 g basis (Scholl & Mansfeld, 1910 ▸). The resulting diol was pyrolyzed with ten times the qu­antity of Cu powder for 45 min at 360 °C under N_2_. Gradient sublimation gave an excellent yield of compound V and a smaller yield of IV, both not free of each other. There were also two major by-products: VI (m.p. >427 °C) and VII (m.p. 426–427 °C). Repeated gradient sublimation and chromatography gave four pure (>99%) products. Besides VI and VII, we eventually identified at least four other by-products, but these were not obtained free of each other.

### 2.2. Spectroscopy

**Figure 3:**
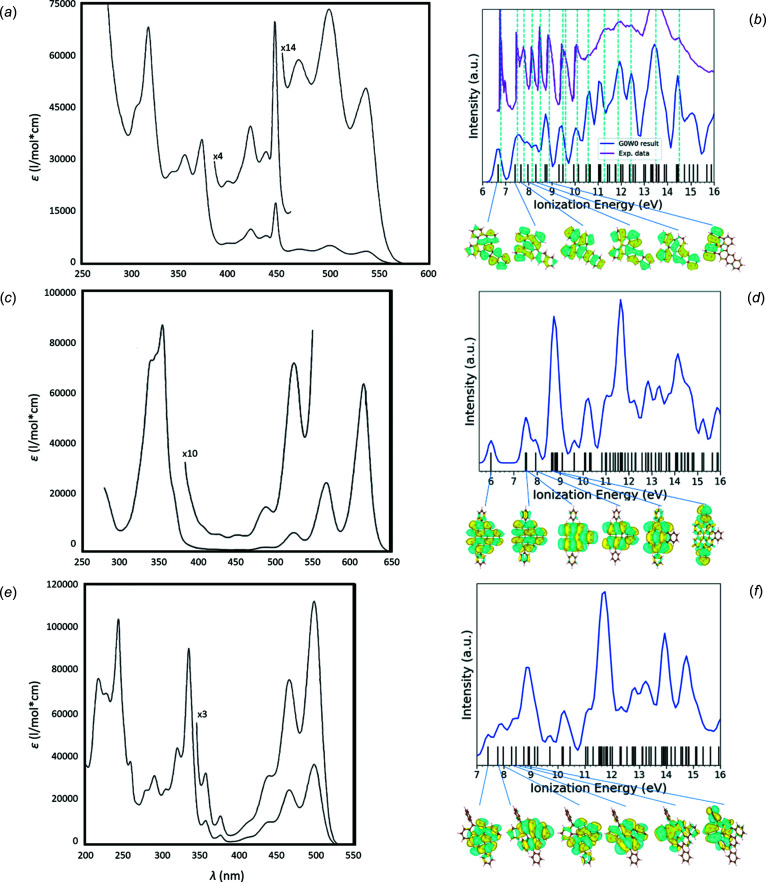
(*a*)/(*c*)/(*e*) UV and (*b*)/(*d*)/(*f*) simulated GW@PBE0 spectra of (*a*)/(*b*) com­pound I, (*c*)/(*d*) com­pound VI and (*e*)/(*f*) com­pound VII. On the UV spectra, numbers indicate zoomed areas. Comparison of the simulated GW@PBE0 spectrum (in blue) with the experimental PE spectrum (in purple) of com­pound I are highlighted (in (*b*)). Frontier orbitals of all three species are also visualized. Gaussian broadening of 0.2 eV was applied to the com­puted ionization energies to simulate the resolution of the experiment.

UV spectroscopic analyses were conducted using an Agilent Cary 300 spectrophotometer. UV spectra were collected at room tem­per­ature using benzene (above 275 nm) or cyclo­hexane (above 200 nm) solvents (Fig. 3 ▸, Figs. S4–S7 and Tables S1–S3). Over 150 UV spectra had to be recorded to ensure the identity and homogeneity of the various fractions. Fractional sublimation was obtained using a Heraeus tube oven ROK 3/30 with a Kelvitron REK 19-20 controller and a melting-point correction. Fluorescence (FL) spectroscopic analyses were carried out with an Aminco–Bowman SPF-500 spectro­fluoro­meter (Table S4). Compound I was insufficiently soluble in cyclo­hexane solvent, so for the determination of reliable extinction coefficients, only benzene was used as solvent. Compound VI is well soluble in benzene and less soluble in cyclo­hexane. Compound VII is well soluble in both solvents. The gas-phase PE spectrum for com­pound I was obtained using a PerkinElmer PS-18 spectrometer with a Helectros Developments photon source (Fig. 3 ▸(*b*)). Samples were also analysed by positive ion mode Matrix-assisted laser desorption/ionization on a Bruker Daltonics ultrafleXtreme II mass spectrometer using Colloidal Graphite as the matrix (Figs. S8–S10).

### 2.3. TEM microscopy and 3D electron diffraction

High-angle annular dark-field scanning transmission electron microscopy (HAADF–STEM) imaging and ED data were recorded with a Zeiss Libra 120 TEM operating at 120 kV and equipped with a LaB_6_ thermionic source. 3D ED was performed in STEM mode after defocusing the beam. Therefore, the beam for the 3D ED experiments was almost parallel when crossing the sample (Köhler illumination). The TEM was set in STEM mode, but the beam, which is normally very convergent in this configuration, was purposely defocused (Benner & Probst, 1994 ▸). The actual beam convergence was 60 µrad. ED patterns were collected with a beam size of about 150 nm in diameter, obtained using a 5 µm C2 condenser aperture. Data were recorded by a single-electron ASI MEDIPIX detector (Nederlof *et al.*, 2013 ▸). An extremely low dose illumination, corresponding to 0.01 e^−^ Å^−2^ s^−1^, was used in order to avoid beam damage. The total dose during data collection depends on many experimental parameters, such as the number of frames, the exposure time and the image tracking mode, and is therefore different for each data collection. A rough estimation of the total dose during a step­wise data collection is in a range of about 1–5 e^−^ Å^−2^. When working with such low doses, the availability of a hybrid-pixel single-electron detector is crucial for acquiring a diffraction pattern with a satisfactory signal-to-noise ratio.

**Figure 4:**
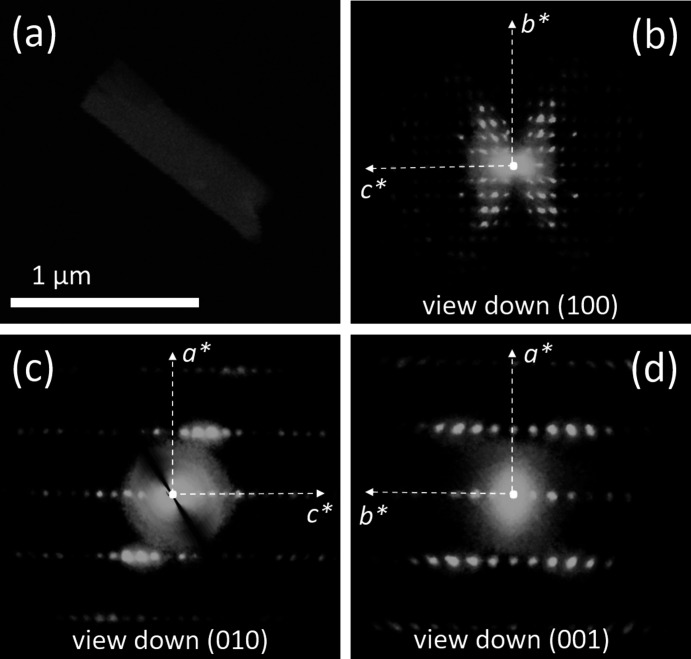
(*a*) HAADF–STEM image of a typical crystal of com­pound I, used for 3D ED data acquisition. (*b*)/(*c*)/(*d*) Reconstructed 3D ED data viewed along the main crystallographic directions. Note that these are projections of a 3D diffraction volume and not 2D oriented ED patterns, and therefore axial extinctions are not visible.

3D ED acquisitions were performed rotating the sample around the TEM goniometer axis in steps of 1°, in total tilt ranges up to 120° (Figs. 4 ▸–6 ▸ ▸). The exposure time per frame was 1 s. The camera length was 180 mm, allowing a resolution in real space of up to 0.7 Å. During the experiment, the beam was precessed around the optical axis by an angle of 1°. Precession was obtained using a Nanomegas Digistar P1000 device. After each tilt, the crystal position was tracked by STEM imaging and a diffraction pattern was acquired. All data acquisitions were performed at room tem­per­ature.

3D ED data were analysed using the software *PETS2.0* (Palatinus *et al.*, 2019 ▸) and *ADT3D* (Kolb *et al.*, 2011 ▸). Structure determination was carried out by standard direct methods (SDM) as implemented in the software *SIR2014* (Burla *et al.*, 2015 ▸), using a fully kinematical approximation (*I_hkl_* proportional to |*F_hkl_* |^2^). Kinematical least-squares structure refinement was performed with the software *SHELXL* (Sheldrick, 2015 ▸) using electron atomic scattering factors. Structures were finally refined considering dynamical effects (Palatinus *et al.*, 2013 ▸, 2015 ▸) using the software *JANA2006* (Petrícek *et al.*, 2014 ▸) and assuming a simple platelet shape.

### 2.4. Computational details

Geometry relaxations of the crystal structures of com­pounds I, VI and VII, and of the single-mol­ecule structure of com­pound I, were conducted using density functional theory (DFT) with the FHI-aims code (Blum *et al.*, 2009 ▸). The Perdew–Burke–Ernzerhof (PBE) (Perdew *et al.*, 1996 ▸, 1997 ▸) generalized gradient approximation was combined with the Tkatchenko–Scheffler (TS) (Tkatchenko & Scheffler, 2009 ▸) pairwise dispersion method. Tight numerical settings and tier 2 basis sets were used. Full unit-cell relaxation was performed until no force com­ponent on any atom exceeded 0.01 eV Å^−1^.

GW+BSE calculations for the crystal structures of com­pounds I, VI and VII were performed using the BerkeleyGW code (Deslippe *et al.*, 2012 ▸). *Quantum ESPRESSO* (Giannozzi, 2009 ▸) was used to com­pute the mean-field eigenvectors and eigenvalues, and to generate the mean-field coarse-grid and fine-grid wave functions with the PBE exchange-correlation functional. For com­pound I, we used a coarse *k*-grid of 8 × 2 × 1 and a fine *k*-grid of 8 × 4 × 2. For com­pound VI, we used a coarse *k*-grid of 4 × 1 × 1 and a fine *k*-grid of 8 × 2 × 2. For com­pound VII, we used a coarse *k*-grid of 2 × 2 × 2 and a fine *k*-grid of 4 × 4 × 4. Troullier–Martins norm-conserving pseudopotentials (Troullier & Martins, 1991 ▸) were used and the kinetic energy cut-off was set at 50 Ry. About 550 unoccupied bands were included in the GW calculation. The static remainder correction was applied to accelerate the convergence with respect to the number of unoccupied states (Deslippe *et al.*, 2013 ▸). The polarizability, inverse dielectric matrix and GW self-energy operator were constructed based on the mean-field eigenvalues and eigenfunctions using the coarse *k*-point settings. Optical properties, including excitation energies, exciton wave functions and absorption spectra were calculated by solving the BSE within the Tamm–Dancoff approximation (TDA). 24 valence bands and 24 conduction bands were included in the BSE calculation (Figs. S11–S16). Taking the full dielectric matrix as input to screen the attraction between the electron (e) and hole (h), the e–h inter­action kernel was constructed on the coarse *k*-point grid. To construct the Bethe–Salpeter Hamiltonian, the GW quasi­particle energies and e–h inter­action kernel calculated with coarse *k*-point settings were inter­polated onto the fine *k*-point grid. The subsequent diagonalization yielded the excitation energies and wave functions. The exciton wave functions of com­pounds I and VI were converged using supercells of 8 × 4 × 2 and 8 × 2 × 2, respectively, based on the criterion proposed in Liu *et al.* (2020*c* ▸). The degree of singlet exciton charge-transfer character (%CT) was calculated by double-Bader analysis (DBA) (Wang *et al.*, 2018 ▸; Liu *et al.*, 2020*c* ▸). The results for rubrene and penta­cene are from Wang *et al.* (2016 ▸), the results for quaterrylene, perylene and tetra­cene are from Wang *et al.* (2018 ▸), the results for anthracene are from Liu *et al.* (2020*c* ▸), and the results for terrylene are from Hall *et al.* (2021 ▸).

GW+BSE calculations for the single mol­ecule of com­pound I were conducted using the FHI-aims code, using augmented tier-2 basis sets (Liu *et al.*, 2020*a* ▸). PBE was used as the DFT starting point. A detailed account of the GW implementation in FHI-aims is provided in Ren *et al.* (2012 ▸) and Caruso *et al.* (2013 ▸). Briefly, the self-energy is first calculated on the imaginary frequency axis and then analytically continued to the real frequency axis. A 16-parameter Pade approximation was used in the analytical continuation (Ren *et al.*, 2012 ▸; Van Setten *et al.*, 2015 ▸). Using the GW quasiparticle energies, BSE calculations were performed to obtain the singlet and triplet excitation energies (Liu *et al.*, 2020*c* ▸). Results for the known TTA chromophores, which we com­pare to com­pound I here, are from Wang *et al.* (2020 ▸), in which the same methodology was used. For GW calculations of the gas-phase PE spectra of I, VI and VII, we used the PBE-based hybrid functional (PBE0) (Adamo & Barone, 1999 ▸) as the mean-field starting point, with tight numerical settings and tier 4 basis sets. This method was benchmarked in Marom *et al.* (2012 ▸) and Knight *et al.* (2016 ▸), and shown to yield spectra in good agreement with experiments for a variety of organic com­pounds. For com­pounds VI and VII, only calculated GW@PBE0 spectra are presented, in the absence of experimental data.

## 3. Results and discussion

### 3.1. Benzo[*e*]di­naphtho­[2,3-*a*;1′,2′,3′,4′-*ghi*]fluoranthene

The mol­ecular conformation of com­pound I was reported as provisional (Clar *et al.*, 1964 ▸). New absorption measurements (Fig. S3) match well with those reported by Clar *et al.* (1964 ▸), apart from the spurious long-wavelength band at 584 nm. The alleged com­position from mass spectrometry was C_34_H_18_ (Fig. S8), also consistent with the related UV spectrum (Fig. 3 ▸(*a*) and Table S1). The occurrence of cyclo­de­hy­drog­en­ation, with the formation of a five-membered ring, is accom­panied by a red shift of the first UV band by 74.5 nm. In particular, the sharp band at about 440 nm is often found in fluoranthene-type PAHs, and never in alternant *cata*- or *peri*-condensed PAHs. The strongest argument in favour of Clar’s assignment comes from the PE spectrum, which fits the calculated one (Clar *et al.*, 1981 ▸). Also, our GW@PBE0 calculation, based on the structure determination by 3D ED data, is in excellent agreement with experimental PE data (Fig. 3 ▸(*b*)). We note that the com­puted ionization energies are vertical values. We do not consider the relaxation of the atomic positions in response to the electronic excitation, vibrational effects, scattering cross-section effects, detector resolution and other sources of noise in the experiment, which may affect the peak breadth and intensity. The com­parison to experiment is focused on peak positions, with the dashed lines at the experimental peak maxima serving as a guide to the eye. Additionally, this spectrum shows an impurity band at 6.95 eV. This peak is not related to the presence of III, which should instead give a band at about 6.67 eV (Clar *et al.*, 1981 ▸).

3D ED data were recorded from three flat crystal fragments of I, with sizes less than 1 µm (Fig. 4 ▸(*a*)). All data sets were consistent with a primitive ortho­rhom­bic unit cell, with parameters *a* = 5.1 (1), *b* = 17.7 (4) and *c* = 23.2 (5) Å (Figs. 4 ▸(*b*)–(*d*)). Such a cell would likely contain four mol­ecules. Upon inspection of the reciprocal space reconstruction, the extinction rules 0*k*0: *k* = 2*n* and 00*l*: *l* = 2*n* were observed. All 3D ED acquisitions miss information about *h*00, because this direction is always parallel to the main surface of the platelet and, standing vertical, cannot be sampled inside the TEM goniometer range. The possible space groups for com­pound I were therefore *P*22_1_2_1_ (No. 18) or *P*2_1_2_1_2_1_ (No. 19), both with multiplicity 4.

Structure solution was performed by SDM using the 3D ED data set with the highest angular range and the greatest resolution. The solution eventually converged in the space group *P*2_1_2_1_2_1_. All but one of the 34 non-H atoms of the mol­ecule were located *ab initio*. The missing C and all H atoms were generated during least-squares refinement against 3D ED data imposing constraints on the aromatic rings. More details about structure determination and refinement are reported in Table S5.

The structure determination of com­pound I allowed for unequivocal confirmation of the mol­ecular model inferred from spectroscopic data, which was previously considered provisional (Clar *et al.*, 1964 ▸). Mol­ecules of I are flat and arranged in four columns per unit cell, which extend along *a*. Inside each column, mol­ecules form a herringbone stack, parallel to one of the four planes of the {3,10,6} family (Figs. 7 ▸(*a*) and 7(*b*)). The inter­molecular distance is about 3.5 Å, similar to that observed in terrylene, another PAH recently solved by the 3D ED method and potentially promising for electronic applications (Hall *et al.*, 2021 ▸).

### 3.2. 7,14-Di­phenyl­naphtho­[1,2,3,4-*cde*]bis­anthene and 7,16-di­phenyl­naphtho­[1,2,3,4-*cde*]heli­anthrene

The two side products VI and VII obtained during the synthesis of IV were separated by repeated gradient sublimation. Compound VI appears as dark-green crystals that do not melt up to 427 °C and shows a deep-red colour in concentrated benzene solution or a carmine colour in dilute solution. While exposed to UV light, it appears violet–blue and has an intense orange–red fluorescence in daylight. Based on mass spectrometry (Fig. S9) and combustion data, VI has the com­position C_46_H_24_. The mol­ecular conformation could not be deduced by spectroscopic data alone and no crystal produced was sufficiently large for single-crystal XRD analysis. In the absence of experimental PE data, we present here the com­puted GW@PBE0 spectra of com­pounds VI and VII (Figs. 3 ▸(*d*) and 3(*f*)).

**Figure 5:**
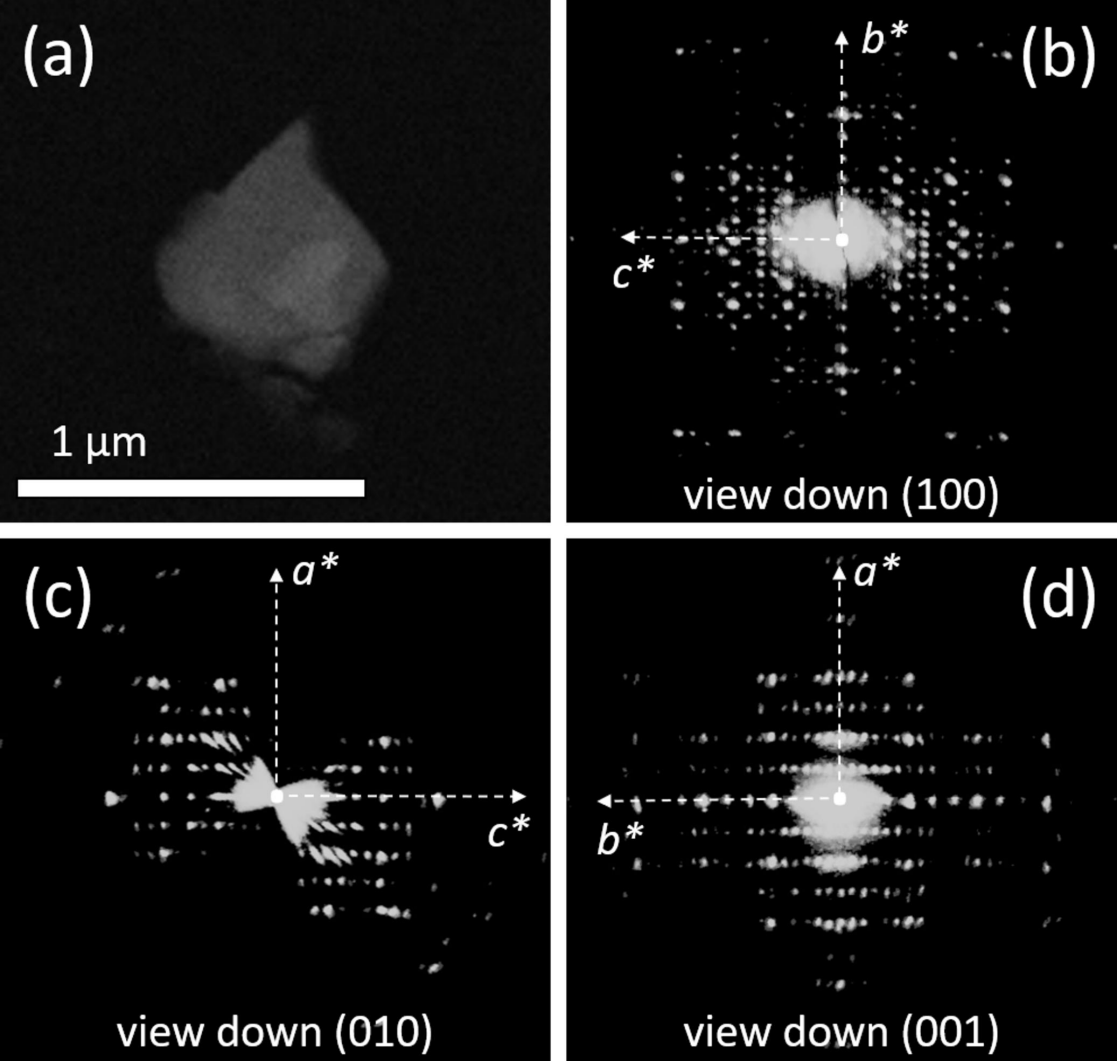
(*a*) HAADF–STEM image of a typical crystal of com­pound VI, used for 3D ED data acquisition. (*b*)/(*c*)/(*d*) Reconstructed 3D ED data viewed along the main crystallographic directions.

3D ED data collected on three VI micrometric crystals (Fig. 5 ▸(*a*)) consistently indicated a primitive ortho­rhom­bic cell with parameters *a* = 9.9 (2), *b* = 26.5 (5) and *c* = 20.7 (4) Å (Figs. 5 ▸(*b*)–(*d*)). Such a cell would likely contain eight VI mol­ecules. Upon inspection of the reciprocal space reconstruction, the extinction rules uniquely indicated the space group *Pbca* (No. 61), with multiplicity 8. Despite the remarkable com­plexity of the mol­ecule, its structure solution was obtained *ab initio* by SDM on the basis of 3D ED data. All 46 non-H atoms of the mol­ecule were located automatically in the first Fourier map, eventually revealing the mol­ecular conformation that could not be obtained by spectroscopic data (Fig. 7 ▸(*c*)). More details about the structure determination and refinement are reported in Table S5.

Mol­ecules of VI are flat and pack as a sandwich herringbone structure parallel to the (102) and (10 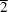 ) planes (Fig. 7 ▸(*d*)). The phenyl groups act as nonplanar satellites of the mol­ecule, forming with it an angle of about 66°. The inter­molecular distance is about 3.5 Å.

Apparently, during the synthesis, phenyl–Li acts as a masked benzyne and connects to the reactive diene positions. A literature search supports the 3D ED result. In fact, the dimesityl analogue of VI was synthesized in an unequivocal manner by benzyne addition to 7,14-bis­(mesit­yl)bis­anthene (Konishi *et al.*, 2013 ▸), when benzyne was liberated *in situ* from *o*-anthranilic acid and isoamyl nitrite. Due to severe twisting about the formal single bond, the phenyl and mesityl groups show little difference; hence, the UV spectra reported by Konishi *et al.* (2013 ▸) agrees with that obtained for com­pound VI (Fig. 3 ▸(*c*)). Compared to IV, lateral annelation shifts the first UV band by 79 nm to the blue (from 684.5 to 613 nm), due to the gain of one Clar sextet. There is a good mirror relationship between the absorption and fluorescence emission, and the Stokes shift is 6 nm.

Compound VII results in ochre crystals with a melting point of 426–427 °C and shows a permanganate colour in solution with benzene or cyclo­hexane. Under UV light it emits a green fluorescence and is moderately stable in daylight. This feature contrasts with the extreme sensitivity of heli­anthrene, whose fluorescence fades in daylight within seconds (Seip & Brauer, 1992 ▸). The combustion and mass spectrometry (Fig. S10) analysis pointed to a com­position of C_46_H_26_. As for com­pound VI, the mol­ecular conformation could not be deduced by spectroscopic data and no crystal was sufficiently large for single-crystal XRD analysis.

**Figure 6:**
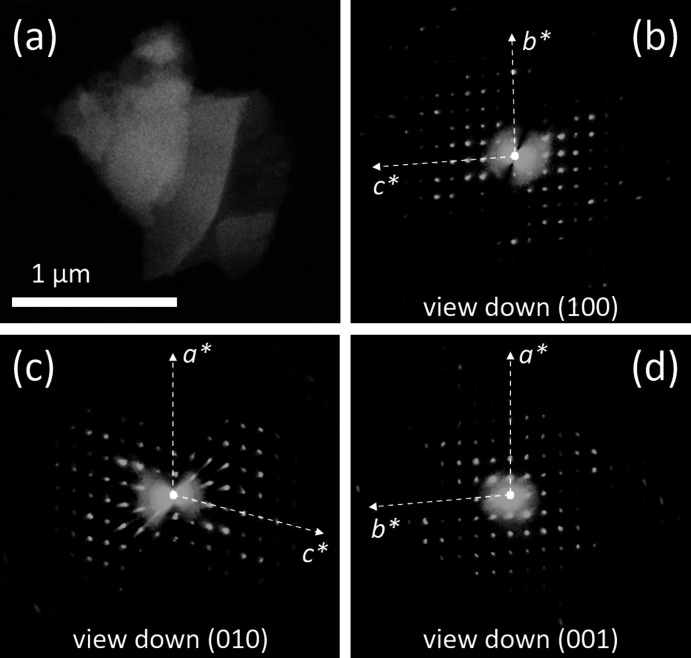
(*a*) HAADF–STEM image of a typical crystal of com­pound VII, used for 3D ED data acquisition. (*b*)/(*c*)/(*d*) Reconstructed 3D ED data viewed along the main crystallographic directions. Note, in the case of com­pound VII, the HAADF image shows more than one single crystal, while the 3D ED data were collected from a single-crystal area (upper right) that appear as thin as the other two com­pounds.

**Figure 7:**
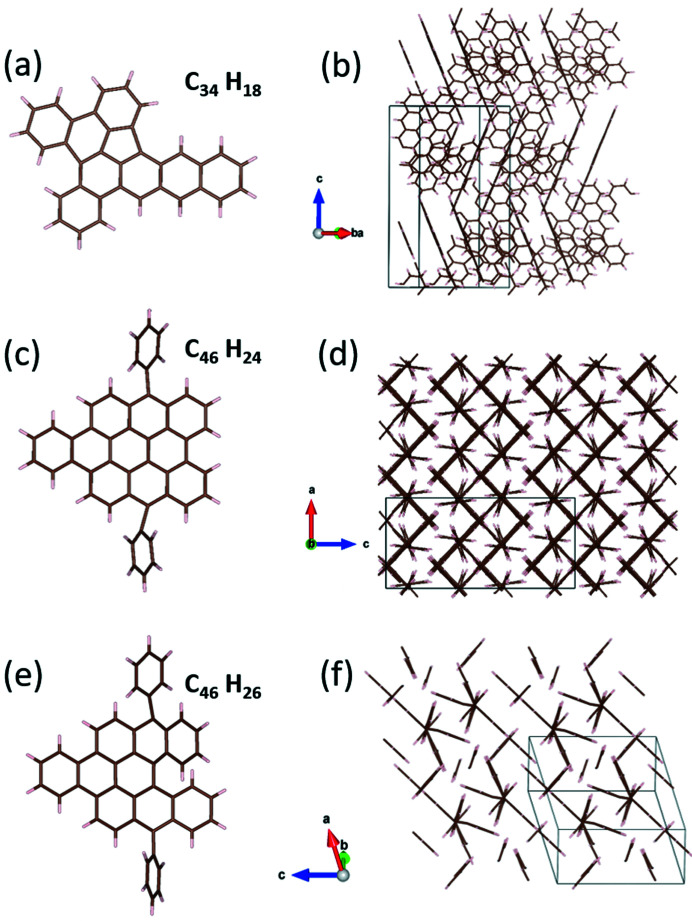
The mol­ecular configuration and crystalline structure of (*a*)/(*b*) com­pound I, (*c*)/(*d*) com­pound VI and (*e*)/(*f*) com­pound VII. C atoms are shown in brown and H atoms are shown in light grey.

3D ED data were recorded from six crystal fragments with a size of less than 1 µm (Fig. 6 ▸(*a*)). All of them delivered a triclinic unit cell with parameters *a* = 10.5 (2), *b* = 11.6 (3), *c* = 12.8 (3) Å, α = 85.3 (5), β = 76.1 (5) and γ = 84.9 (5)° (Figs. 6 ▸(*b*)–(*d*)). No possibility of cell centring was envisaged, and no extinction rule pointing to higher symmetry was recognised. Such a cell would conveniently host only two VII mol­ecules. Structure solution of this com­pound was indeed obtained *ab initio* by SDM in the space group *P* 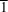 (No. 2). All 46 non-H atoms of the mol­ecule were automatically spotted in the first Fourier map, allowing also, in this case, the mol­ecular conformation to be established, which could not be obtained by spectroscopic data (Fig. 7 ▸(*e*)). More details about the structure determination and refinement are reported in Table S5.

The two terminal phenyl rings in VII act again as nonplanar satellites, forming an angle of about 65° with the main part of the mol­ecule. Their presence partially breaks the aromaticity of the heli­anthrene group. The nonplanarity of VII allows for both C—H⋯π and π–π inter­actions, leading to the reduction of symmetry down to triclinic. No herringbone motif is present (Fig. 7 ▸(*f*)).

In the corresponding UV spectrum, a first sharp band was observed at 501 nm (Fig. 3 ▸(*e*)). Consistent with the 3D ED results, in going from V to VII, there is a blue shift by 79 nm due to the gain of one Clar sextet in the mol­ecule. The Stokes shift is 16.5 nm (Fig. S7, Tables S2 and S3).

### 3.3. Electronic properties

Having definitively resolved the mol­ecular and crystal structures of com­pounds I, VI and VII by 3D ED, we are now able to use com­puter simulations to assess their potential usefulness for photovoltaic applications. In particular, we are inter­ested in discovering new materials capable of undergoing singlet fission (SF), the down-conversion of one singlet exciton into two triplet excitons (Smith & Michl, 2010 ▸, 2013 ▸; Monahan & Zhu, 2015 ▸; Michl, 2019 ▸), and its reverse process, triplet–triplet annihilation (TTA), the up-conversion of two triplet excitons into one singlet exciton (Baldo *et al.*, 2000 ▸; Schmidt & Castellano, 2014 ▸; Schulze & Schmidt, 2015 ▸). Both of these processes can be harnessed to reduce losses and thus increase the conversion efficiency of solar cells. In a typical solar cell, the absorption of one photon produces one charge carrier. The absorption of a high-energy photon generates a highly excited singlet exciton, which subsequently thermalizes to the lowest excited state, such that the excess photon energy is lost to heat. SF can be potentially utilized to convert that excess photon energy into an additional charge carrier by generating two triplet excitons from one high-energy singlet exciton. Another source of losses in solar cells is that photons with energies below the gap of the absorber cannot be absorbed and their energy is lost. TTA can be utilized to convert two low-energy photons into an additional charge carrier. Hence, supplementing the traditional absorber with SF and TTA materials can significantly enhance the efficiency of solar cells by reducing losses at both the high end and the low end of the solar photon energy spectrum. SF and TTA materials are rare because few chromophores meet the stringent requirements for the excited-state energetics, as detailed below. Most of the known and predicted SF and TTA chromophores are PAHs, mainly acene and perylene derivatives. Therefore, we evaluate the PAHs studied here for these purposes.

**Figure 8:**
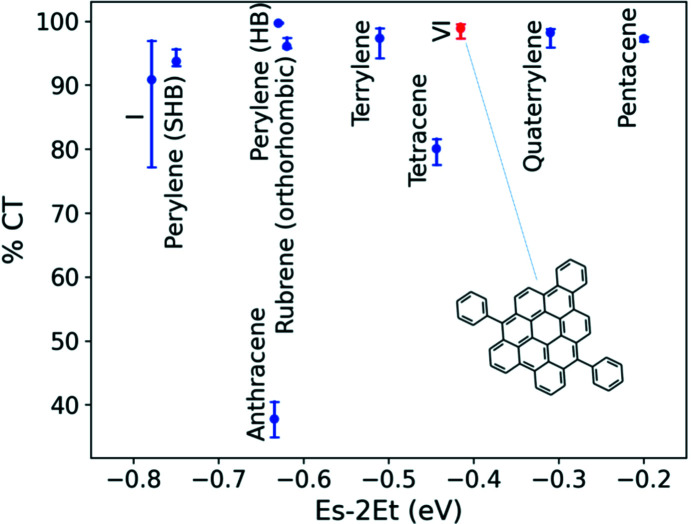
A two-dimensional descriptor for assessing SF candidates. The thermodynamic driving force for SF (*E*_s_ − 2*E*_t_) is displayed on the *x* axis and the singlet exciton charge-transfer character (%CT) is displayed on the *y* axis. The error bars correspond to the range of %CT values obtained by using different hole positions when performing the double-Bader analysis. Compounds I and VI (the latter highlighted in red) are com­pared to some acene and rylene phases.

To assess the potential of mol­ecular crystals to undergo SF in the solid state, we consider a two-dimensional descriptor (Wang *et al.*, 2018 ▸; Liu *et al.*, 2020*b* ▸,*c* ▸), as shown in Fig. 8 ▸. The primary descriptor, displayed on the *x* axis, is the thermodynamic driving force for SF, which is the difference between the singlet exciton energy and twice the triplet exciton energy (*E*_s_ − 2*E*_t_). A high driving force indicates that a material is likely to undergo SF with a high rate. However, an overly high driving force would lead to losses in solar energy conversion. Therefore, it has been suggested that materials with *E*_s_ ≃ 2*E*_t_ may be preferable (Wu *et al.*, 2014 ▸). Owing to the approximations used in GW+BSE calculations, the values of *E*_s_ − 2*E*_t_ are systematically underestimated. Hence, we restrict the discussion to qualitative com­parisons between materials.

The secondary descriptor, displayed on the *y* axis, is the degree of charge-transfer character (%CT) of the singlet exciton wave function. Exciton wave functions have two spatial variables corresponding to the electron and hole probability distributions. If the hole is located on one mol­ecule, the degree of charge-transfer character corresponds to the probability of finding the electron on other mol­ecules. This descriptor is motivated by the growing body of experimental evidence for the involvement of an inter­mediate charge-transfer state in the SF process (Monahan & Zhu, 2015 ▸; Chan *et al.*, 2013 ▸; Kim *et al.*, 2019 ▸; Miyata *et al.*, 2019 ▸; Margulies *et al.*, 2017 ▸). A singlet exciton with a high degree of charge-transfer character is thought to be favourable for SF (Monahan & Zhu, 2015 ▸; Sharifzadeh *et al.*, 2013 ▸, 2015 ▸; Broch *et al.*, 2018 ▸; Hart *et al.*, 2018 ▸). To evaluate %CT, we use double-Bader analysis (DBA), an extension of the Bader charge-partitioning scheme, to exciton wave functions with two spatial variables. The %CT is calculated by performing nested sums over the electron distributions obtained for different hole positions within a mol­ecule. The error bars correspond to the range of %CT values obtained for different hole positions within the double-Bader analysis (Wang *et al.*, 2018 ▸; Liu *et al.*, 2020*c* ▸).

In Fig. 8 ▸, com­pounds I and VI are com­pared to perylenes and acenes with respect to these two descriptors. Penta­cene has the highest SF driving force of the materials shown here and is known to undergo fast SF with a high triplet yield (Chan *et al.*, 2011 ▸; Wilson *et al.*, 2011 ▸). SF in tetra­cene is experimentally known to be slightly endoergic (Tomkiewicz *et al.*, 1971 ▸; Grumstrup *et al.*, 2010 ▸; Burdett *et al.*, 2010 ▸; Chan *et al.*, 2012 ▸; Burdett & Bardeen, 2012 ▸, 2013 ▸; Arias *et al.*, 2016 ▸). Compound VI has a somewhat higher SF driving force, as well as a higher singlet exciton CT character than tetra­cene. Based on this, it may be a promising candidate for inter­molecular SF in the solid state. Compound I has a lower SF driving force than anthracene and perylene, whose derivatives are known to undergo TTA (Renaud *et al.*, 2013 ▸; Jiang *et al.*, 2013 ▸; Eaton *et al.*, 2013 ▸; Mirjani *et al.*, 2014 ▸; Renaud & Grozema, 2015 ▸; Le *et al.*, 2016 ▸; Würthner *et al.*, 2016 ▸). Therefore, it may be able to undergo TTA. The com­puted singlet and triplet excitation energies of com­pound VII are 2.90 and 2.00 eV, respectively. It is thus not a likely candidate for either SF or TTA; therefore, it is not shown in Figs. 8 ▸ and 9 ▸.

**Figure 9:**
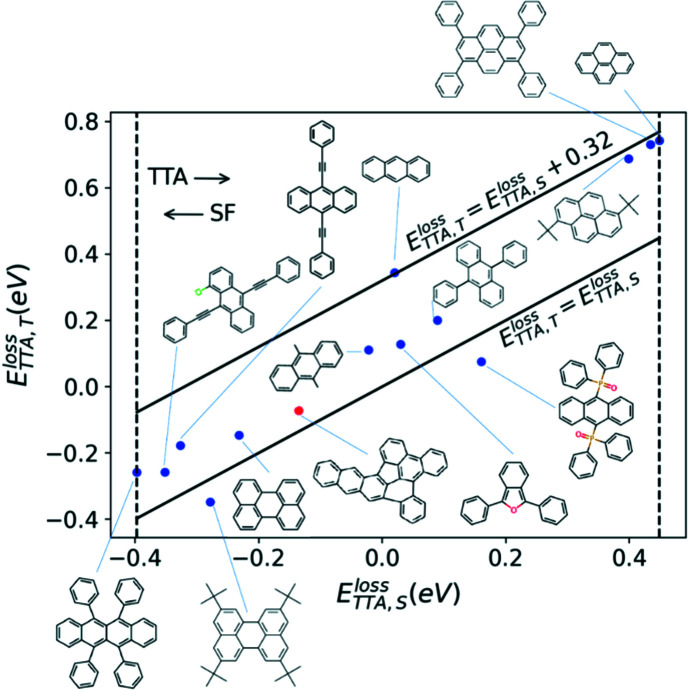
Assessment of com­pound I (highlighted in red) as a TTA candidate, com­pared to several known TTA chromophores. The energy release in the singlet pathway, 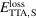 = 2*T*_1_ − *S*_1_, is plotted on the *x* axis and the energy release in the com­peting triplet pathway, 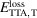 = 2*T*_1_ − *T*_2_, is plotted on the *y* axis. The dashed line on the left correspond to the 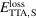 of rubrene and the dashed line on the right corresponds to the 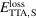 of pyrene. The region delineated by the two diagonal lines is where most experimentally known TTA chromophores are found in Wang *et al.* (2020 ▸).

To further assess the potential of com­pound I to undergo TTA, we com­pare its excited-state energetics as an isolated mol­ecule to several chromophores experimentally known to undergo TTA (Fig. 9 ▸). The com­parison is based on the criteria proposed in Wang *et al.* (2020 ▸). The primary criterion for a chromophore to undergo TTA is that the energy release in the singlet pathway, 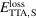 = 2*T*_1_ − *S*_1_, plotted on the *x* axis, should be positive to provide thermodynamic driving force, but not overly large to avoid significant energy losses. As discussed in detail in Wang *et al.* (2020 ▸), GW+BSE@PBE systematically underestimates 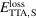 . Therefore, we assess new chromophores based on a com­parison with known TTA chromophores, similar to our procedure for assessing SF candidates. In rubrene, TTA is experimentally known to be approximately isoergic (Cheng *et al.*, 2010 ▸). Therefore, we consider the 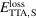 of rubrene as the lower limit for the singlet pathway to be open. Mol­ecules with a smaller 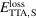 than rubrene may be more likely to undergo SF than TTA. This is indicated by the left dashed vertical line in Fig. 9 ▸. Of the experimentally known TTA chromophores calculated in Wang *et al.* (2020 ▸), pyrene has the highest 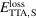 . We then consider pyrene as the upper limit, above which the energy loss is too high for efficient TTA. This is indicated by the right vertical dashed line in Fig. 9 ▸.

The main process com­peting with TTA is the combination of two triplet excitons into a higher-energy triplet state, *T*_2_, which decays nonradiatively. Hence, a secondary criterion for the quantum yield of TTA to be as high as possible is for the energy release in the triplet pathway, 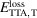 = 2*T*_1_ − *T*_2_, to be as small as possible com­pared to 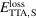 . In Wang *et al.* (2020 ▸), we have found that most of the experimentally known TTA chromophores fall into the range between 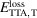 = 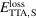 and 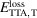 = 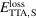 + 0.32 eV. This is indicated by the two diagonal lines in Fig. 9 ▸. Compound I (highlighted in red) is well within this range, between perylene and an anthracene derivative. Therefore, based on energetic considerations, com­pound I may be a promising candidate for TTA. We note, however, that favourable energetics are a necessary but not sufficient condition for good TTA chromophores (Wang *et al.*, 2020 ▸).

## 4. Conclusions

Organic syntheses typically yield several side products, which are generally discarded even if their qu­antity amounts to 20–50% of the total. Such side products are often unwanted. Moreover, the assessment of their potential use is com­plicated by the difficulty of their characterization. Spectroscopic data may be insufficient to unambiguously determine the mol­ecular conformation, especially when mol­ecules are com­plex or the presence of more than one species is suspected. Powder X-ray diffraction requires a sufficient amount of material and may suffer from peak overlap, in particular when the phase of inter­est cannot be properly purified, its unit-cell parameters are long and/or small crystal size causes severe peak broadening. In these cases, 3D electron diffraction emerges as a promising technique for conclusive determination of the mol­ecular conformation and crystal structure, as we have demonstrated here for benzo[*e*]di­naphtho­[2,3-*a*;1′,2′,3′,4′-*ghi*]fluoranthene (com­pound I), 7,14-diphenyl-naphtho­[1,2,3,4-*cde*]bis­anthene (com­pound VI) and 7,16-di­phenyl­naphtho­[1,2,3,4-*cde*]heli­anthrene (com­pound VII).

Once the structure is determined, com­puter simulations may be used to predict the electronic and optical properties of side products, in particular when the amount of material initially produced is insufficient for detailed experimental characterization. This can help to assess the potential usefulness of side products for various applications. In some cases, unintended side products may turn out to be as useful as the main reaction products. For example, the second system analysed here com­prises four related mol­ecules. Of these, IV exhibits exceptional optical properties (Gorelenko *et al.*, 1977 ▸) and V found practical application as a pigment for visible-light-sensitive actinometers (Brauer *et al.*, 1983 ▸). Based on the many-body perturbation theory simulations presented here, com­pounds I and VI may be useful for photovoltaic applications. Compound I may exhibit triplet–triplet annihilation, which may enable harvesting photons with energies below the gap of the absorber in a solar cell. Compound VI may exhibit inter­molecular singlet fission in the solid state, which may enable the harvesting of two charge carriers from one high-energy photon in a solar cell. Compound VII is a wide-gap insulator.

We hope that the results reported in this article will inspire others to pursue further characterization of organic side products by 3D ED and com­puter simulations. This may lead to the discovery of potentially useful com­pounds for various applications. Thus, 3D ED is a promising new avenue for enriching our knowledge of organic synthetic routes and exploiting side products that would otherwise be discarded.

## Supplementary Material

Crystal structure: contains datablock(s) I, VI, VII. DOI: 10.1107/S205225252201154X/ur5001sup1.cif

Structure factors: contains datablock(s) I. DOI: 10.1107/S205225252201154X/ur5001Isup2.hkl

Structure factors: contains datablock(s) VI. DOI: 10.1107/S205225252201154X/ur5001VIsup3.hkl

Structure factors: contains datablock(s) VII. DOI: 10.1107/S205225252201154X/ur5001VIIsup4.hkl

Supporting information. DOI: 10.1107/S205225252201154X/ur5001sup5.pdf

CCDC references: 2223426, 2225208, 2225209
