# Supplementary material for: True mol­ecular conformation and structure determination by three-dimensional electron diffraction of PAH by-products potentially useful for electronic applications
Source: IUCrJ. 2023 Jan 1;10(Pt 1):131–42. doi: 10.1107/S205225252201154X (PMC9812223; doi:10.1107/S205225252201154X)
Supplement: Supplementary file 5 [file m-10-00131-sup5.pdf]

IUCrJ

Volume 10 (2023)

Supporting information for article:

True molecular conformation and structure determination by three-dimensional electron diffraction of PAH by-products potentially useful for electronic applications

Iryna Andrusenko, Charlie L. Hall, Enrico Mugnaioli, Jason Potticary, Simon R. Hall, Werner Schmidt, Siyu Gao, Kaiji Zhao, Noa Marom and Mauro Gemmi

Synthesis details

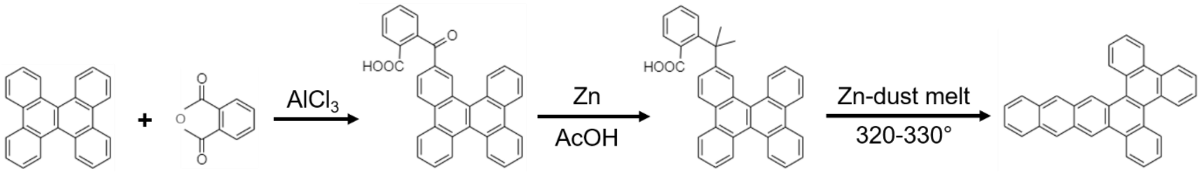

Figure S1. An alternative synthesis of II, namely by Friedel-Crafts reaction of dibenzo(*g*,*p*)chrysene and phthalic acid anhydride. For steric reasons, phthalic acid anhydride attacks dibenzo(*g*,*p*)chrysene most likely at the 2- or 3-position (IUPAC numbering, shown is 2-position). Stepwise reduction with Zn dust gives II exclusively.

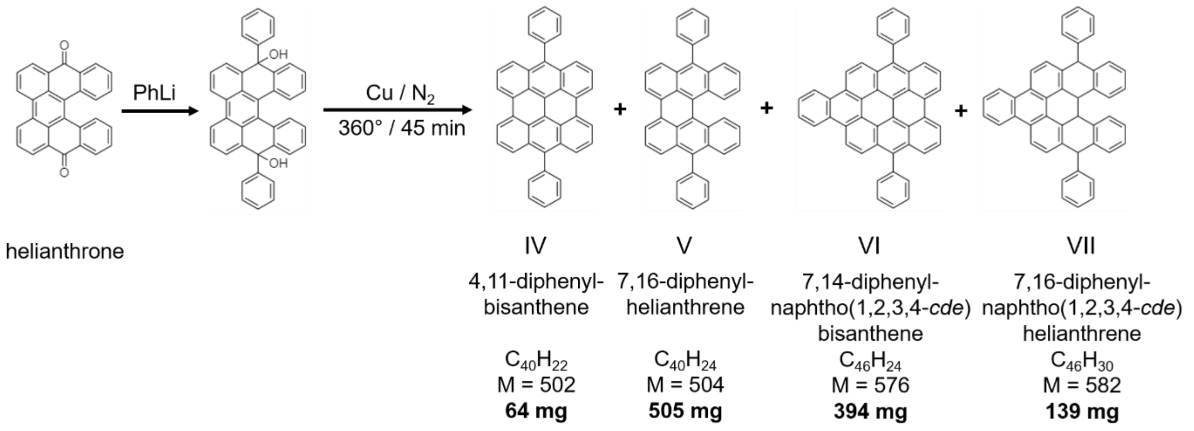

Figure S2. A stepwise model of the synthesis of 4,11-diphenyl-bisanthene (IV), 7,16-diphenyl-helianthrene (V), 7,14-diphenyl-naphtho(1,2,3,4-*cde*)bisanthene (VI) and 7,16-diphenyl-naphtho(1,2,3,4-*cde*)helianthrene (VII). For each product of the reaction, formula, molecular weight (M) and yield (in bold) are reported.

Spectroscopy details

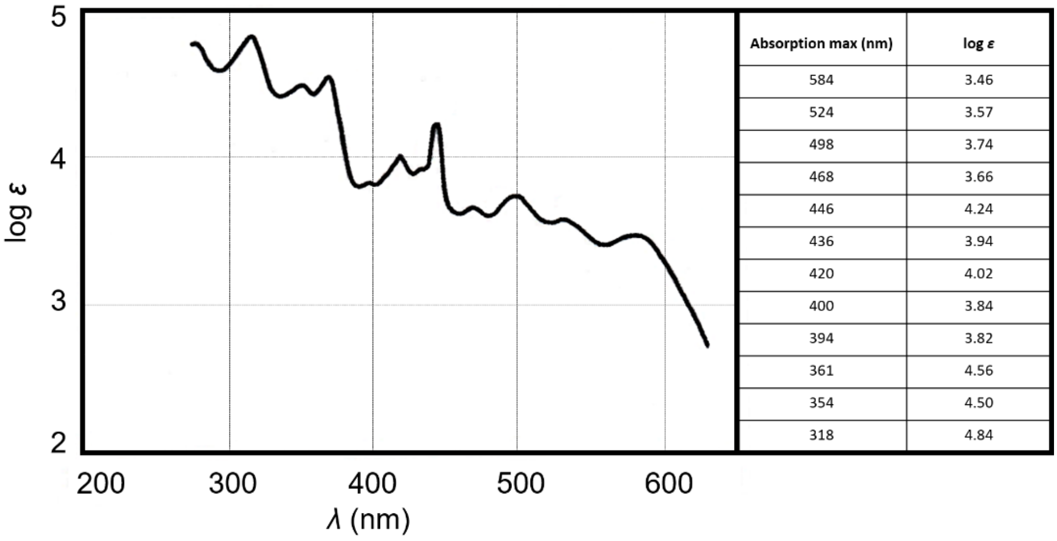

Absorption max (nm)	log *ε*
584	3.46
524	3.57
498	3.74
468	3.66
446	4.24
436	3.94
420	4.02
400	3.84
394	3.82
361	4.56
354	4.50
318	4.84

Figure S3. Absorption spectrum of compound I.

Table S1. Details of UV spectrum of compound I.

***λ* (nm)**	***ε* (l/mol*cm)**
535.5	3530
499.0	5110
469.0	4090
445.0	17000
435.5	7890
420.0	9690
397.5	5830
371.0	35000
353.5	30700

341.5	26000
317.5	66700
277.5	61500

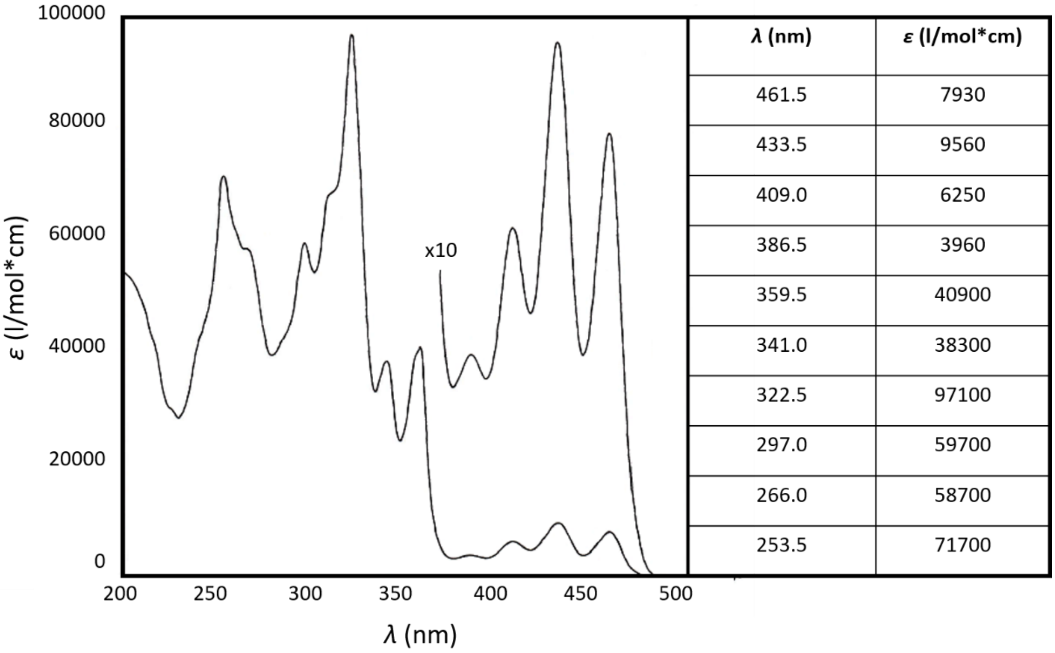

λ (nm)	ε (l/mol*cm)
461.5	7930
433.5	9560
409.0	6250
386.5	3960
359.5	40900
341.0	38300
322.5	97100
297.0	59700
266.0	58700
253.5	71700

Figure S4. UV spectrum of compound II.

The molecular structure of compound III is suspected (Clar *et al.*, 1964). According to UV spectroscopic observation (Fig.S5), the vibrational spacings (1289/1040/1376 cm^{-1}) in the first band are not in line with an alternant *peri*-condensed PAH, viz. 1400 cm^{-1} and the intensity pattern is unusual. It appears that two electronic transitions are involved, one beginning at 489 nm, the other at 439 nm. This casts doubts about the homogeneity of the substance. Some previously calculated values (Clar *et al.*, 1981) indicate that the 489/469 nm peaks are authentic, and that the 439/414 nm peaks are due to an unknown impurity that is in line with Clar’s annellation rules. Unfortunately, due to lack of material, a PE spectrum could not be acquired.

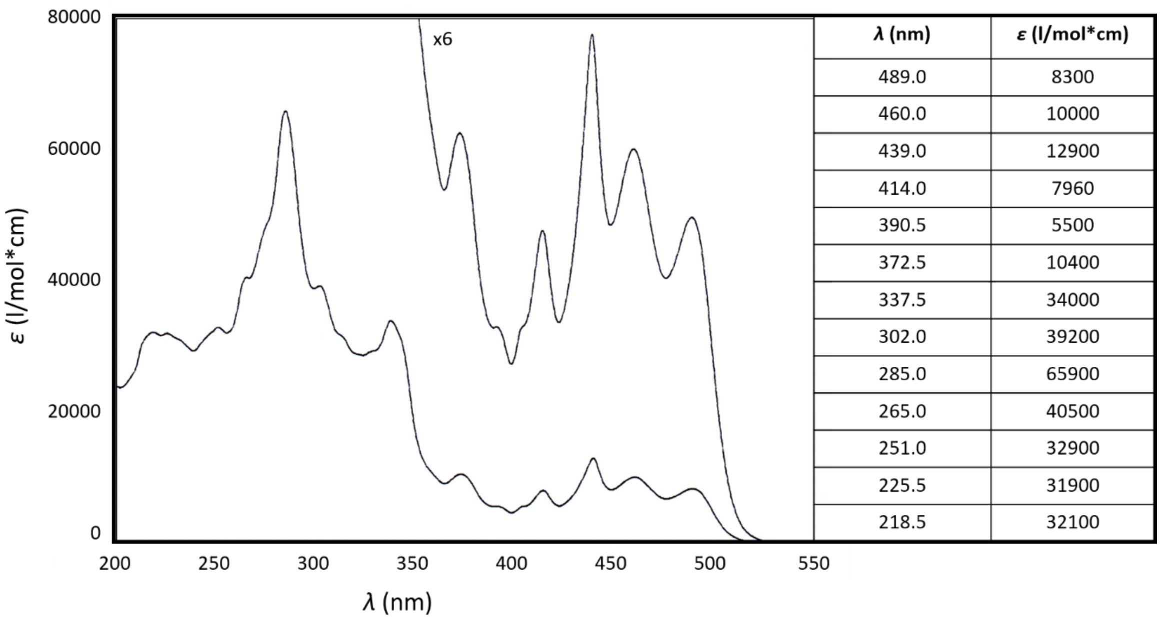

λ (nm)	ε (l/mol*cm)
489.0	8300
460.0	10000
439.0	12900
414.0	7960
390.5	5500
372.5	10400
337.5	34000
302.0	39200
285.0	65900
265.0	40500
251.0	32900
225.5	31900
218.5	32100

Figure S5. UV spectrum of compound III.

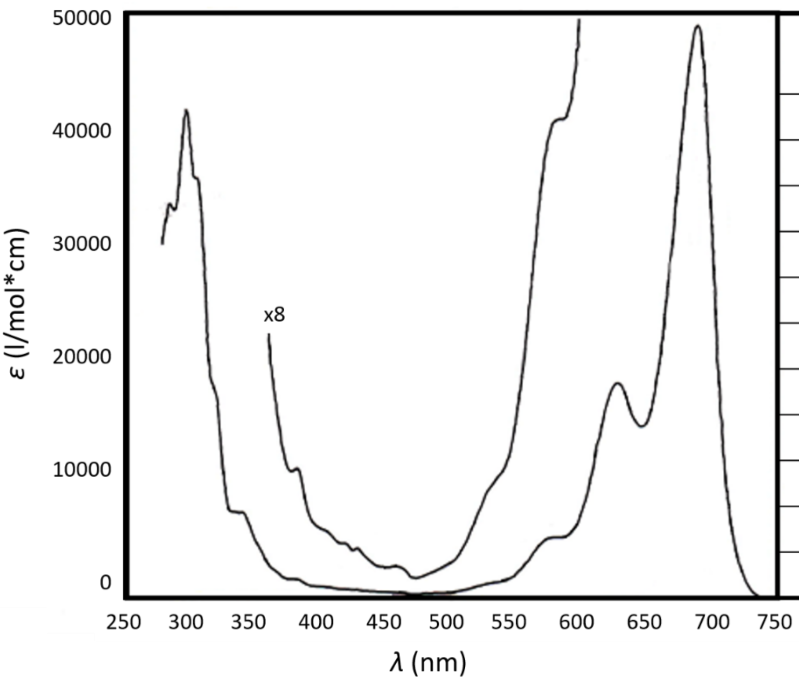

λ (nm)	ε (l/mol*cm)
684.5	49100
625.0	18300
579.0	5130
457.0	320
427.5	510
381.0	1350
339.0	6980
333.0	7030
302.5	35600
294.0	41500
282.0	33400

Figure S6. UV spectrum of compound IV.

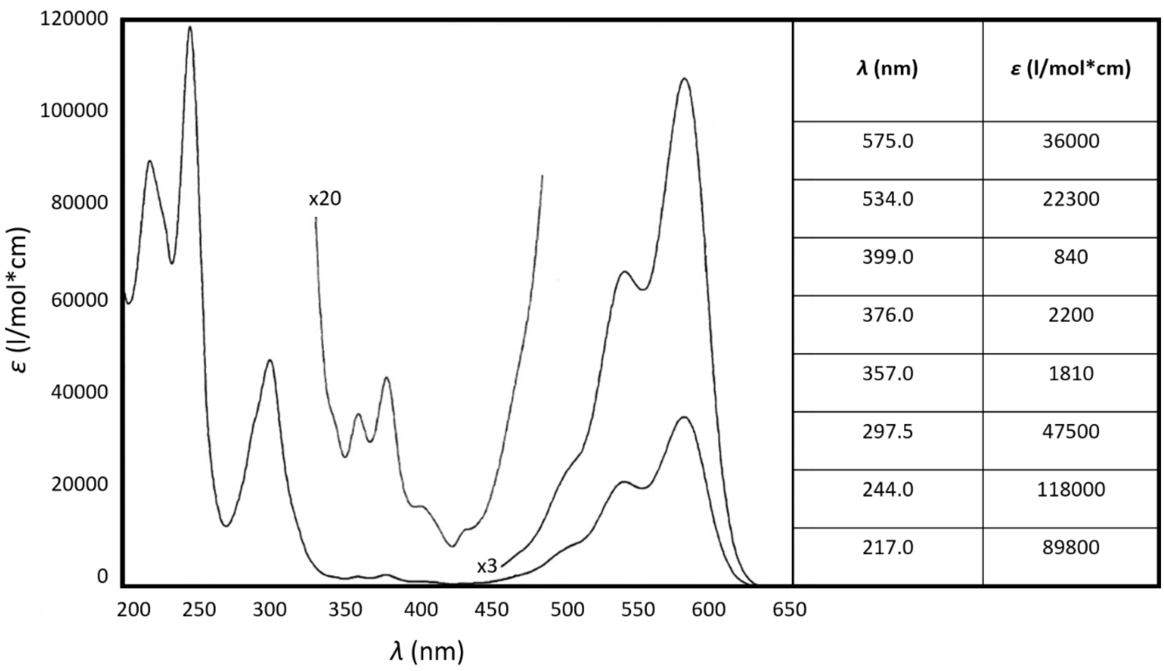

λ (nm)	ε (l/mol*cm)
575.0	36000
534.0	22300
399.0	840
376.0	2200
357.0	1810
297.5	47500
244.0	118000
217.0	89800

Figure S7. UV spectrum of compound V.

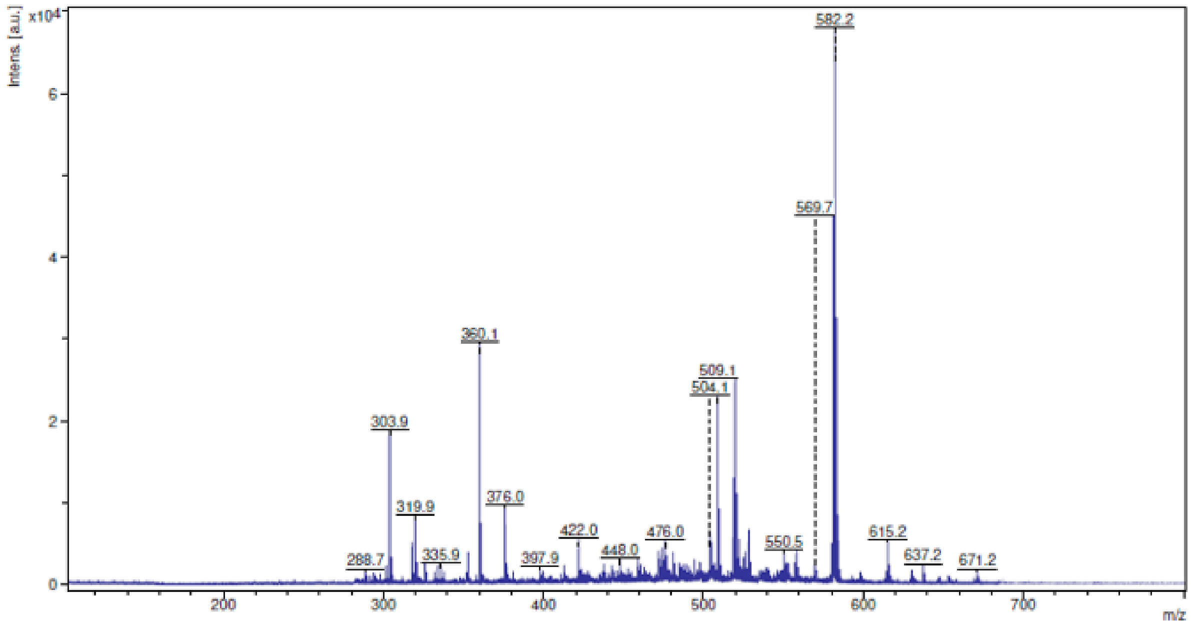

Figure S8. Mass spectrometry of compound I.

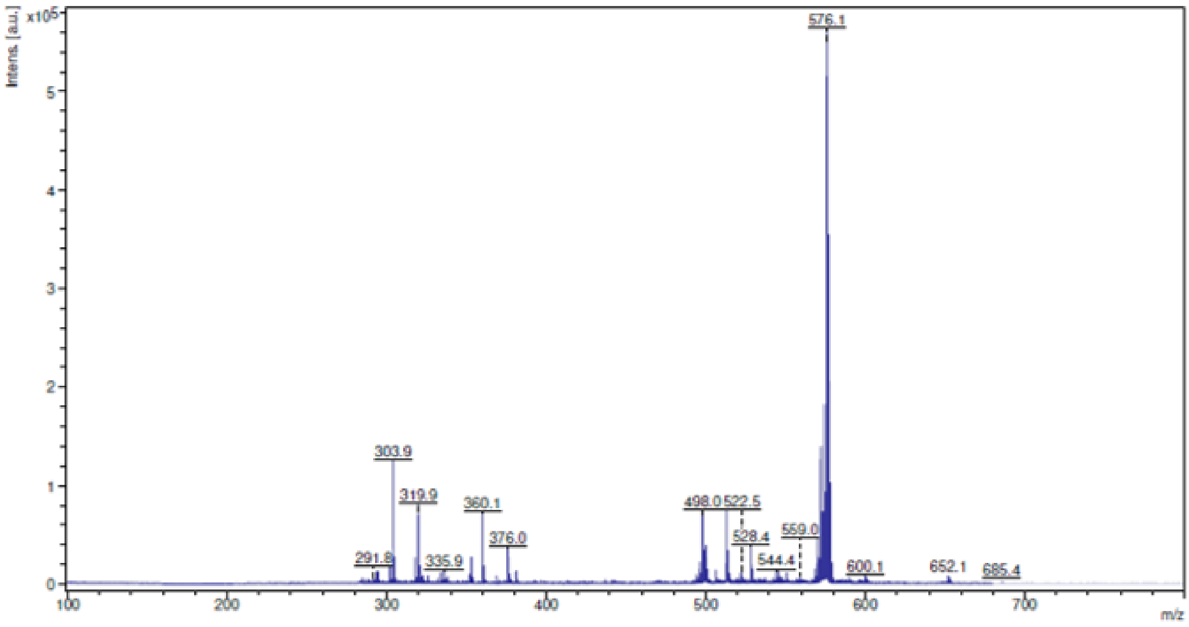

Figure S9. Mass spectrometry of compound VI.

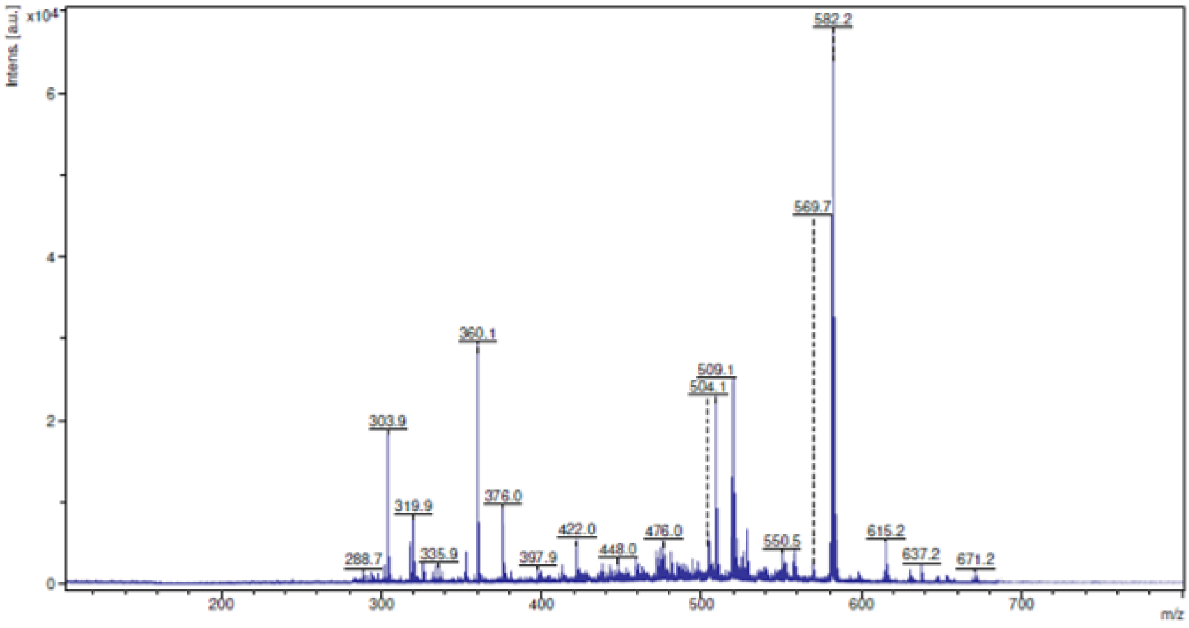

Figure S10. Mass spectrometry of compound VII.

Table S2. Details of UV spectrum of compound VI.

**λ (nm)**	**ε (l/mol*cm)**
613.0	64500
565.0	26600
523.0	7230
486.5	1710
451.0	630
423.0	660
353.0	86800
338.0	73200

Table S3. Details of UV spectrum of compound VII.

**λ (nm)**	**ε (l/mol*cm)**
496.0	37500
464.0	25500
438.0	10700
375.0	4440
355.5	11100
334.0	90700
319.0	44900
304.0	25600
289.5	31800
279.0	25100
258.5	38400
243.0	104000
227.0	69800
217.0	76600

Table S4. Details of FL spectra of compounds IV, V, VI, VII.

***λ* (nm)**	***Rel. Int.***
**Compound (IV)**	
696.0	1.00
**Compound (V)**	
602.5	1.00
**Compound (VI)**	
672.5	0.14
619.0	1.00
**Compound (VII)**	
544.0	0.45
512.5	1.00

A list of correspondent solvents used for obtaining spectroscopic data:

compound I – benzene;

compound II – cyclohexane;

compound III – cyclohexane;

compound IV – benzene;

compound V – cyclohexane;

compound VI – benzene;

compound VII – cyclohexane.

Structure solution details

Table S5. Selected parameters from structures determination and refinement.

	**Compound (I)**	**Compound (VI)**	**Compound (VII)**
Crystallographic information			
Asymmetric unit content	$C_{34}H_{18}$	$C_{46}H_{24}$	$C_{46}H_{26}$
Z	4	8	2
Space group	$P2_12_12_1$	*Pbca*	$P\bar{1}$
a (Å)	5.1(1)	9.9(2)	10.5(2)
b (Å)	17.7(4)	26.5(5)	11.6(3)
c (Å)	23.2(5)	20.7(4)	12.8(3)
α (°)	90	90	85.3(5)
β (°)	90	90	76.1(5)
γ (°)	90	90	84.9(5)
Volume ($Å^3$)	2094(73)	5431(188)	1504(60)
Ab-initio structure determination by *SIR2014*			
Tilt range (°)	120	95	120
Data resolution (Å)	0.9	0.9	0.9
Sampled reflections (No.)	8413	16107	6039
Independent reflections (No.)	1543	3456	3078
Independent reflection coverage (%)	86	89	71
Global thermal factor U_{iso} ($Å^2$)	0.05269	0.00149	0.02813
R_{int} (%)	43.37	58.53	21.99
R_{SIR} (%)	28.74	37.13	29.11
Kinematical refinement by *SHELXL*			
R_{int} (%)	32.67	64.71	19.23
No. of total reflections	2647	3456	3078
No. of reflections > 4σ	1191	1541	1528
$R1_{4\sigma}$ (%)	27.52	43.32	34.36
$R1_{all}$ (%)	38.78	53.62	43.98

Goodness-of-fit	2.058	2.299	2.841
Dynamical refinement by *JANA2006*			
g_{max} (Å^{-1})	1.5	1.0	1.5
Maximal S_g^0 (matrix) (Å^{-1})	0.01	0.01	0.01
Maximal S_g^0 (refine) (Å^{-1})	0.1	0.1	0.1
RS_g	0.4	0.4	0.4
No. of integration steps (precession)	96	64	64
No. of zones	77	68	101
No. of reflections	8927	9870	8265
No. of reflections > 3σ	1138	1463	1315
Calculated thickness (Å)	563	652	537
R_{obs} (%)	17.15	24.88	16.23
wR_{all} (%)	20.05	28.35	19.60
Goodness-of-fit	1.82	2.11	1.84

Electronic properties details

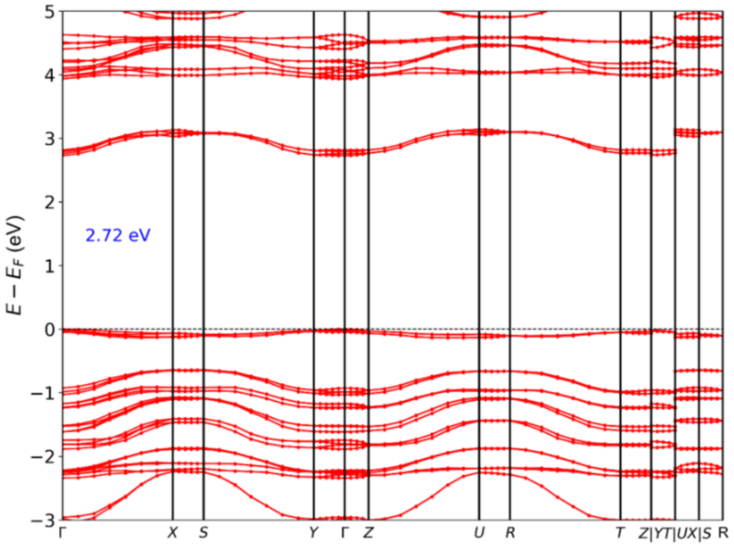

Figure S11. GW@PBE quasiparticle band structure of compound I.

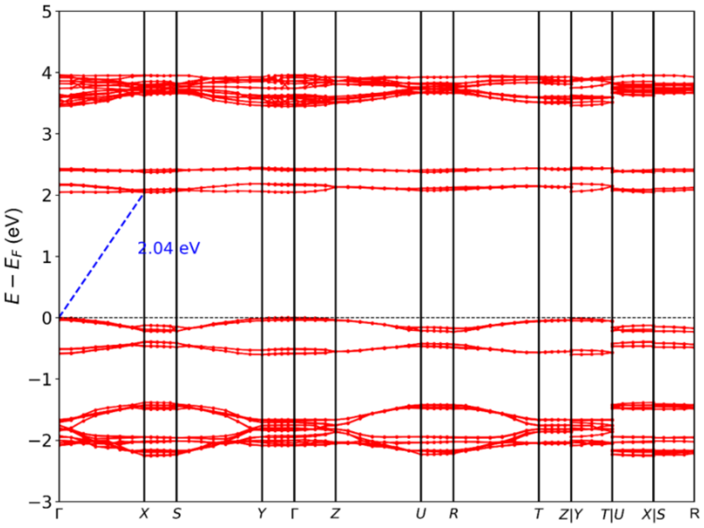

Figure S12. GW@PBE quasiparticle band structure of compound VI.

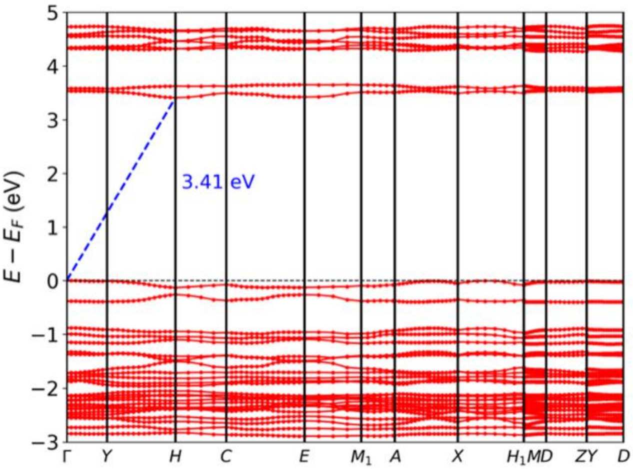

Figure S13. GW@PBE quasiparticle band structure of compound VII.

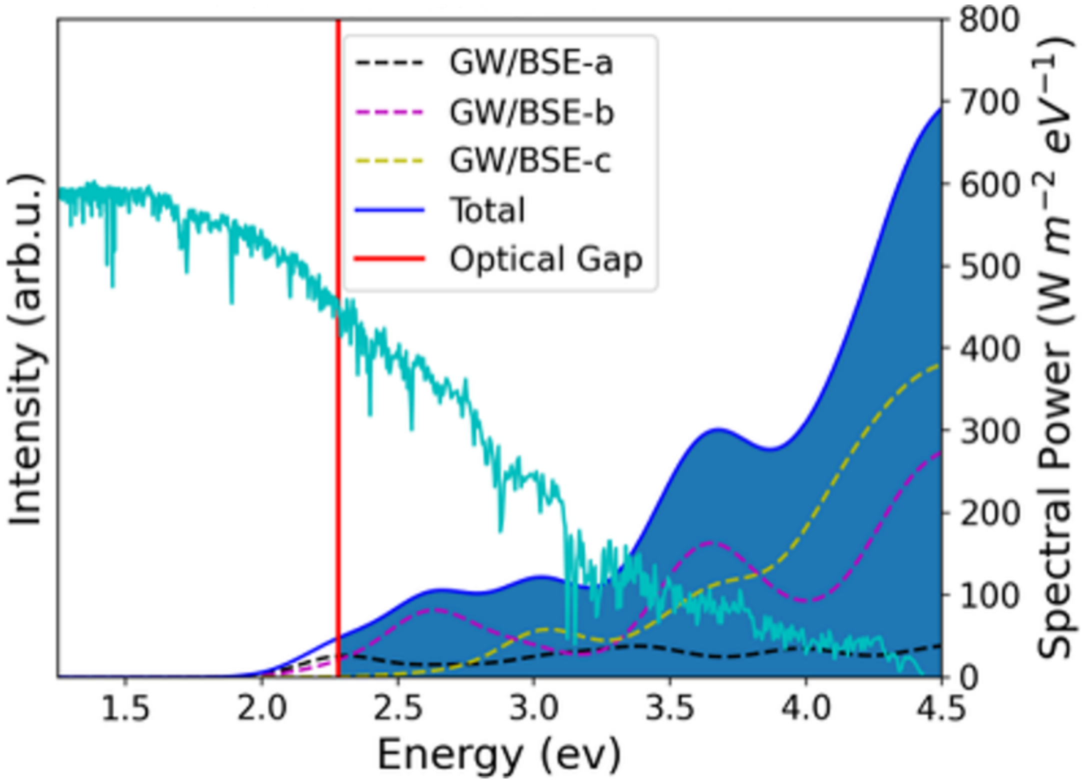

Figure S14. GW+BSE@PBE absorption spectrum of compound I for light polarized along the three crystal axes. The solar spectrum is also shown.

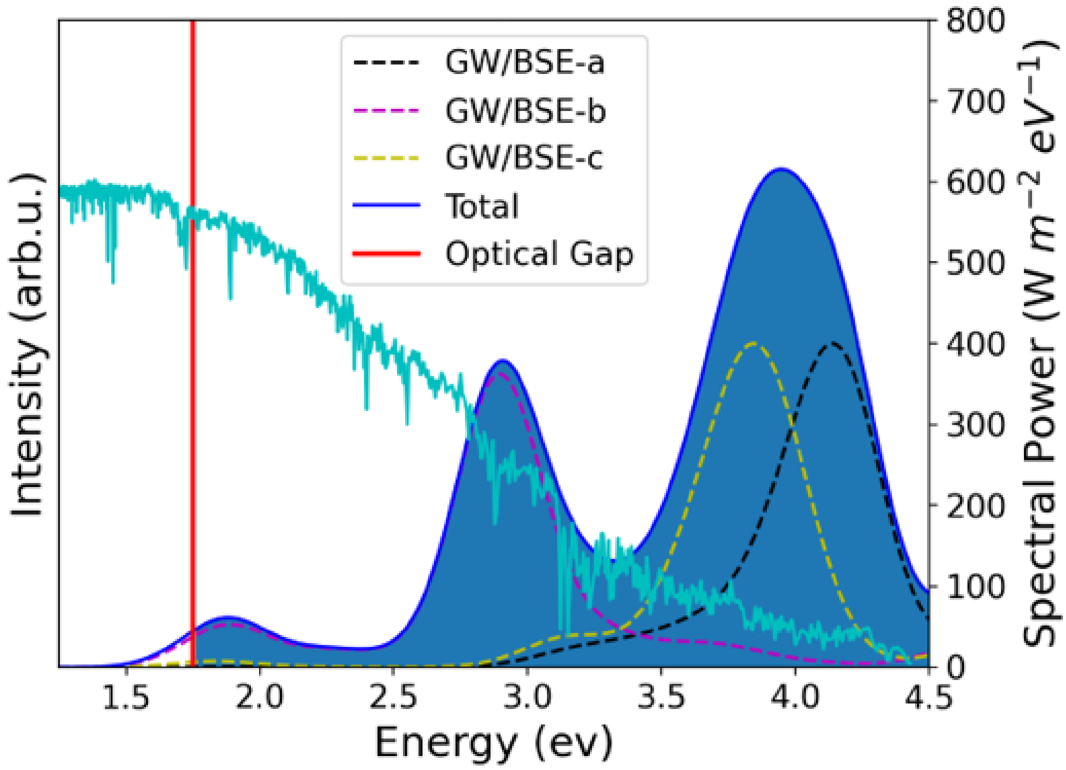

Figure S15. GW+BSE@PBE absorption spectrum of compound VI for light polarized along the three crystal axes. The solar spectrum is also shown.

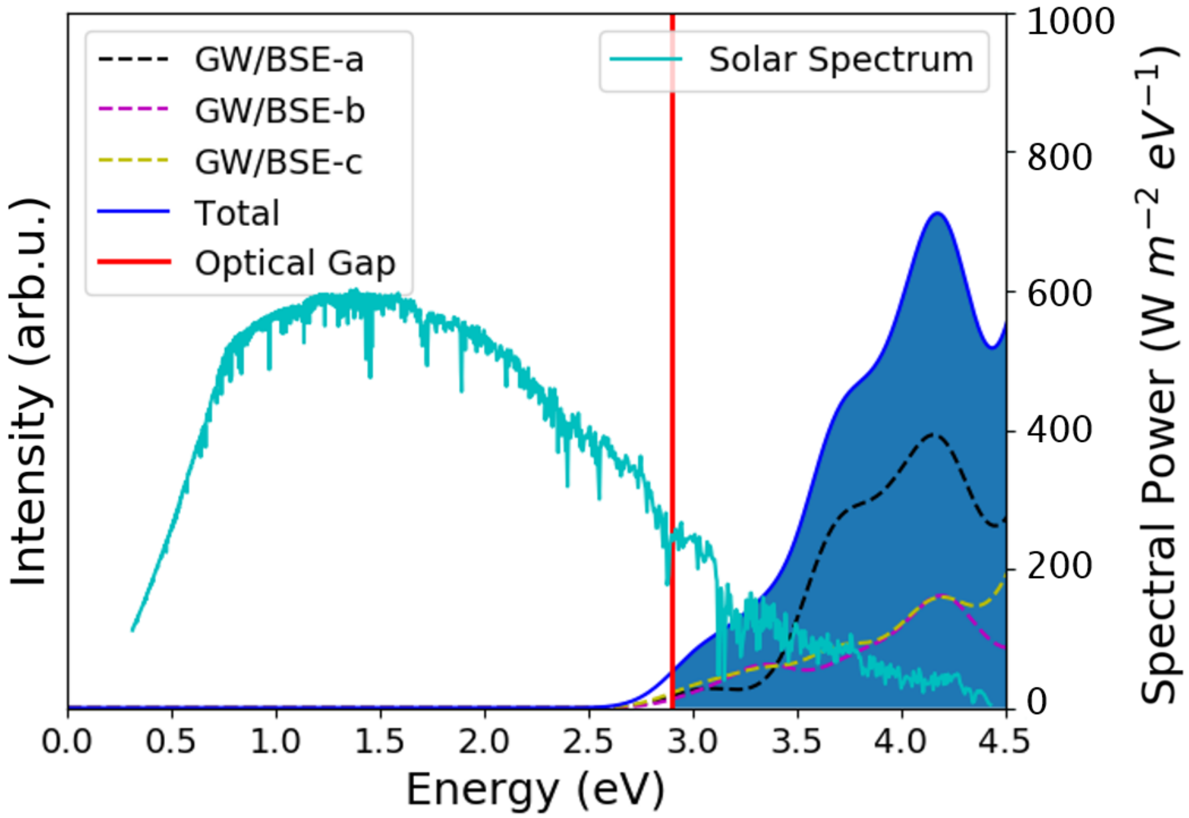

Figure S16. GW+BSE@PBE absorption spectrum of compound VII for light polarized along the three crystal axes. The solar spectrum is also shown.
